# Increased *AR* expression in castration-resistant prostate cancer rapidly induces AR signaling reprogramming with the collaboration of EZH2

**DOI:** 10.3389/fonc.2022.1021845

**Published:** 2022-11-03

**Authors:** Maryam Labaf, Muqing Li, Lily Ting, Breelyn Karno, Songqi Zhang, Shuai Gao, Susan Patalano, Jill A. Macoska, Kourosh Zarringhalam, Dong Han, Changmeng Cai

**Affiliations:** ^1^ Center for Personalized Cancer Therapy, University of Massachusetts Boston, Boston, MA, United States; ^2^ Department of Mathematics, University of Massachusetts Boston, Boston, MA, United States; ^3^ Department of Biology, University of Massachusetts Boston, Boston, MA, United States; ^4^ Department of Medicine, Vanderbilt University, Nashville, TN, United States; ^5^ Department of Cell Biology and Anatomy, New York Medical College, Valhalla, NY, United States; ^6^ Department of Biochemistry and Molecular Biology, New York Medical College, Valhalla, NY, United States

**Keywords:** androgen, androgen receptor, AR, prostate cancer, androgen-deprivation therapy, CRPC, EZH2, DNA damage repair

## Abstract

Elevated androgen receptor (AR) expression is a hallmark of castration-resistant prostate cancer (CRPC) and contributes to the restoration of AR signaling under the conditions of androgen deprivation. However, whether overexpressed AR alone with the stimulation of castrate levels of androgens can be sufficient to induce the reprogramming of AR signaling for the adaptation of prostate cancer (PCa) cells remains unclear. In this study, we used a PCa model with inducible overexpression of AR to examine the acute effects of AR overexpression on its cistrome and transcriptome. Our results show that overexpression of AR alone in conjunction with lower androgen levels can rapidly redistribute AR chromatin binding and activates a distinct transcription program that is enriched for DNA damage repair pathways. Moreover, using a recently developed bioinformatic tool, we predicted the involvement of EZH2 in this AR reprogramming and subsequently identified a subset of AR/EZH2 co-targeting genes, which are overexpressed in CRPC and associated with worse patient outcomes. Mechanistically, we found that AR-EZH2 interaction is impaired by the pre-castration level of androgens but can be recovered by the post-castration level of androgens. Overall, our study provides new molecular insights into AR signaling reprogramming with the engagement of specific epigenetic factors.

## Introduction

Androgen receptor (AR) plays a pivotal role in prostate cancer (PCa) initiation and progression (1). AR transcription activity can be blocked by androgen deprivation therapies (ADTs), which include second-generation AR signaling inhibition (ARSi) agents, such as abiraterone and enzalutamide (2, 3), but tumors eventually adapt to these treatments with a more aggressive form (castration-resistant prostate cancer, CRPC) (4). Importantly, these AR signaling inhibition treatments often lead to significantly increased *AR* gene expression that can happen rapidly through a negative-feedback mechanism or by *AR* gene amplification or alterations of *AR* regulatory elements (5–7). While it is relatively clear that *AR* gene overexpression is an important factor in driving CRPC progression (8), it is still unclear how *AR* overexpression can engage with epigenetic pathways to reprogram AR signaling and allow PCa cells to adapt to the various treatments.

EZH2, a key component of polycomb complex (PRC2), functions as a lysine methyltransferase to methylate histone 3 lysine 27 (H3K27) (9) and is overexpressed in many types of cancers including PCa, particularly CRPC (10). While EZH2 is well known for its transcription repression activity by producing methylated H3K27, which are repressive histone marks, it can also act as a transcription activator independent of its PRC2 activity in PCa (11). One important function is that EZH2 can act as an AR coactivator by directly interacting with AR and transcriptionally regulating expressions of AR target genes (11, 12). A recent study has also shown that EZH2 is highly involved in activating DNA repair machinery in response to genotoxic stress in CRPC (13). Therapeutically, EZH2 inhibitors in combination with AR signaling inhibition treatments are currently being tested for mCRPC in clinical trials (e.g., CELLO-1 trial).

In this study, we have generated a lentiviral stable cell line with doxycycline-inducible overexpression of *AR*. Treating with the combination of doxycycline and different doses of androgens, this system can provide *AR* ^low^/DHT^high^ or *AR* ^high^/DHT^low^ status to mimic the levels of the receptor and ligand under pre- and post-castration conditions. Our data demonstrated that induced overexpression of AR can rapidly alter the AR chromatin binding, which results in specific activation of a subset of genes that are highly enriched for DNA replication and damage repair pathways. Using a recently developed transcription factor predication algorism, we have revealed the involvement of EZH2 in this reprogramming of AR and identified a 68-gene signature to predict activities of AR/EZH2 co-regulated gene transcription in CRPC. Our data further revealed that the expression of these genes is significantly increased in CRPC tumor samples in comparison with primary PCa samples and is associated with poor clinical outcomes. Moreover, we also found that AR-EZH2 interaction is impaired by high-dose androgen stimulation but maintained by low-dose androgens. Together, our data provide a global view of AR signaling reprogramming in CRPC driven by increased *AR* expression and decreased ligand levels and suggest the engagement of EZH2 for this activity of AR.

## Materials and methods

### Cell lines and cell culture

LNCaP and C4-2 cell lines were purchased from ATCC, authenticated every six months using short tandem repeat (STR) profiling, and frequently tested for mycoplasma contamination using the MycoAlert mycoplasma detection kit (Lonza). LNCaP cells were cultured in RPMI with 10% FBS (fetal bovine serum) and C4-2 cells were cultured with 2% FBS plus 8% charcoal-stripped FBS (CSS). LNCaP stable cell line overexpressing tetracycline-regulated AR (LNCaP-tet-AR) was generated by lentiviral infection of pLIX_403 tetracycline-inducible lentiviral vector with wild-type full-length AR, using the Gateway Technology with Clonase II (Invitrogen, Cat# 12535-029). LNCaP-tet-AR cells were cultured in RPMI with 10% tetracycline-free FBS. For androgen stimulation assays, cells were grown to 50-60% confluence in culture medium containing 5% CSS for 3 days (d) and then treated with DHT or inhibitors.

### Immunoprecipitation and immunoblotting

For immunoprecipitation assays (IP), cells were lysed in Triton Lysis buffer and treated with protein inhibitor cocktails (Thermo Fisher Scientific). Lysates were then immunoprecipitated with anti-AR (06-680, Millipore) antibody and proteins were eluted by boiling in Laemmli buffer with 5% beta-mercaptoethanol. For immunoblotting, cells were lysed with RIPA buffer containing protease inhibitor cocktail. Anti-BRD4 (ab128874, Abcam), anti-EZH2 (39901, Active Motif), anti-AR (06-680, Millipore), and anti-GAPDH (ab8245, Abcam) were used as primary antibodies.

### Quantitative real-time RT-PCR

RNA was isolated from cells with TRIzol reagent (Invitrogen), Quantitative real-time PCR was performed using Fast 1-step Mix (Thermo Fisher Scientific) on QuantStudio 3 PCR machine. All TaqMan primer/probe sets were predesigned and purchased from Thermo Fisher Scientific, and PCR results were normalized to GAPDH.

### Cell proliferation assay

LNCaP-tet-AR cells were pre-cultured with 5% CSS. Cells were then plated into 12-well plates supplemented with/out 0.25 mg/ml doxycycline. After 1-2d, cells were treated with DHT for 24 hours (h) and then treated with DMSO, olaparib (S1060, Selleckchem), cisplatin (S1166, Selleckchem), or GSK126 (S7061, Selleckchem) for 0-6d. Cells were trypsinized, collected, and counted by countess II automated cell counter (Invitrogen).

### Chromatin immunoprecipitation (ChIP) and ChIP-seq analysis

For the preparation of ChIP, dispensed cells were formalin-fixed, lysed, and sonicated to break the chromatin into 200–300 bp fragments (500-800 bp fragments for ChIP-qPCR assays), followed by immunoprecipitation with ChIP grade antibodies: anti-AR (ab108341, Abcam), anti-EZH2 (39901, Active Motif), or Rabbit/Mouse IgG (Millipore). Primer sequences for ChIP-qPCR (using SYBR green) are: BRCA1-ARE forward, 5’-AATGGGGATGACAAGACAGG-3’; BRCA1-ARE reverse, 5’-AGGGGTGGACCCTACATTATC-3’; BRCA2-ARE forward, 5’-TGTAAGCAGATTTGTTGAATATTTG-3’; BRCA2-ARE reverse,5’-ATCCAGGAGGCATTGCATAA-3’; BRIP1-ARE forward, 5’- TCAGCACCACATCGCACT-3’; BRIP1-ARE reverse, 5’-CACACTGAGAGATTCTGGTACG-3’; MCM2-ARE forward, 5’-GTACAGTGGCACGCAGCTC-3’; MCM2-ARE reverse, 5’-AGGGCGGAGCTTTGTGTAT-3’.

ChIP-seq libraries were constructed using the SMARTer ThruPLEX DNA-Seq Prep Kit (Takara Bio USA). Next-generation sequencing (51nt, Paired-end) was performed using Illumina NextSeq 2000 Genome Analyzer. ChIP-sequencing reads were mapped to the hg19 human genome using BWA (version 0.7.5a) with aln and samse sub-commands (-l 32 -q 5 -k 2) (14). Samtools (version 0.0.19) was used to convert sam files to bam format. The significance of enriched ChIP regions was evaluated by using MACS3 (version 3.0.0a6) (15) with FDR *q*-value = 0.05, fix-bimodal and extend size set as 200 using the narrow peak caller. The bedGraph files containing signal per million reads produced from MACS were converted into bigwig files using UCSC tools (version 372). The R package ChIPpeakAnno (version 3.26.4) was used to analyze peak intervals and determine the overlapped regions. Venn diagrams were generated using VennDiagram (version 1.7.3) R package. The signals associated with genomic regions were visualized by using computeMatrix and plotHeatmap tools from deepTools (version 3.0.2). computeMatrix with reference-point mode was used to calculate scores for each genomic region, and plotHeatmap was used to create a heatmap for scores associated with genomic regions. MACS-generated peaks were ranked by their score (-10 x Log_10_(*q*-value)) and the top 5,000 peaks were imported to SeqPos in Galaxy/Cistrome (16) for motif enrichment analysis. To identify significantly enriched motifs under a given condition, motifs were ranked by z-score, and the difference in rank was plotted on a waterfall plot. Binding and Expression Target Analysis was performed by BETA (version 1.0.7) (17) to integrate ChIP-seq with differential gene expression to predict direct targets. Peak interval files from MACS and differential expression results from edgeR were used as inputs.

### RNA-seq analysis

RNA from cell lines was extracted by using Rneasy Kit (QIAGEN). RNA-Seq library was prepared using TruSeq Stranded RNA LT Kit (Illumina). Sequencing was performed on NextSeq 2000 Illumina Genome Analyzer. The single-end reads were processed by FastQC (version 0.11.6) (18) and aligned by STAR (version 2.5.3a) to the human Ensemble genome (Ensembl, GRCh37) with all default parameters (19). featureCounts (version 1.6.2) from Subread package was used to assign sequence reads to the genomic features. All gene counts were processed with R package edgeR (3.24.1) to evaluate the differential expression using the glmQLFIT to fit the count data and by applying Benjamini–Hochberg false discovery rate (FDR)-adjusted *P* value (20). The expression values were centered and scaled across samples. The pre-ranked gene lists were used to conduct Gene Set Enrichment Analysis (GSEA) by using R package fgsea (version 1.18.022.0) (21) with msigdbr Hallmark (version 7.5.1). The top pathways with normalized enrichment scores (NES) ranked by adjusted *P* value were plotted for visualization. R package gprofiler2 (version 0.2.1) (22) was utilized to perform the gene ontology for differentially expressed genes. The significantly enriched pathways were filtered by FDR *q*-value < 0.05.

### Signature analysis

The process to identify the *AR* ^high^/DHT^low^ and *AR* ^low^/DHT^high^ gene signatures is as follows: (i) The genes for both conditions were filtered by fold-change > 1.5, FDR < 0.05 and direct targets of peaks were generated by ChIP-seq; (ii) these two filtered gene sets were overlapped and the unique genes for each group were selected; (iii) For each unique gene sets, the genes that have fold-change difference of greater than 1.5 to other groups, were selected. The public RNA-seq data of siEZH2 in LNCaP-ABL cells (GSE39452) (11) were retrieved and re-analyzed for differentially expressed genes with fold-change > 1.5 with FDR < 0.05. Gene overlaps were performed by R package UpsetR (1.4.0). To determine the enrichment of AR/EZH2 target gene set over the sample population, we applied the non-parametric and unsupervised gene set enrichment analysis using GSVA (version 1.40.1) R package. The z-scores relative to all the samples were used as input. The genes of these signatures were listed in **Supplementary Table 1**.

### Transcription factor prediction analysis

The differential gene expressions under *AR* ^low^/DHT^high^ and *AR* ^high^/DHT^low^ were defined as the fold-change > 1.5 with FDR < 0.05 in comparison with vehicle. The genes were ranked by fold-change and FDR and fed to Causaul Inference Engine (CIE) (23) to identify the active transcriptional regulators using the ChIP-Atlas prostate cell-line database with Fisher-exact test to perform the enrichment test. The output network was filtered by transcription factors (TFs) and adjusted *P*-value < 0.05.

### Statistical analysis

Data in bar graphs represent mean ± SD of at least 3 biological repeats. Statistical analysis was performed using Student’s *t*-test by comparing treatment versus vehicle control or otherwise as indicated. *P*-value < 0.05 was considered to be statistically significant (* for *P* < 0.05, ** for *P <*0.01, ***for *P* < 0.001, ns for P > 0.05). Box plots of the signature score and gene expression were compared using the Wilcoxon test for comparison between the two conditions. All statistical analyses and visualization were performed with R (version 4.1.1) unless otherwise specified.

## Results

### AR chromatin binding is altered in *AR* ^high^/DHT^low^ cells

**Figure 1:**
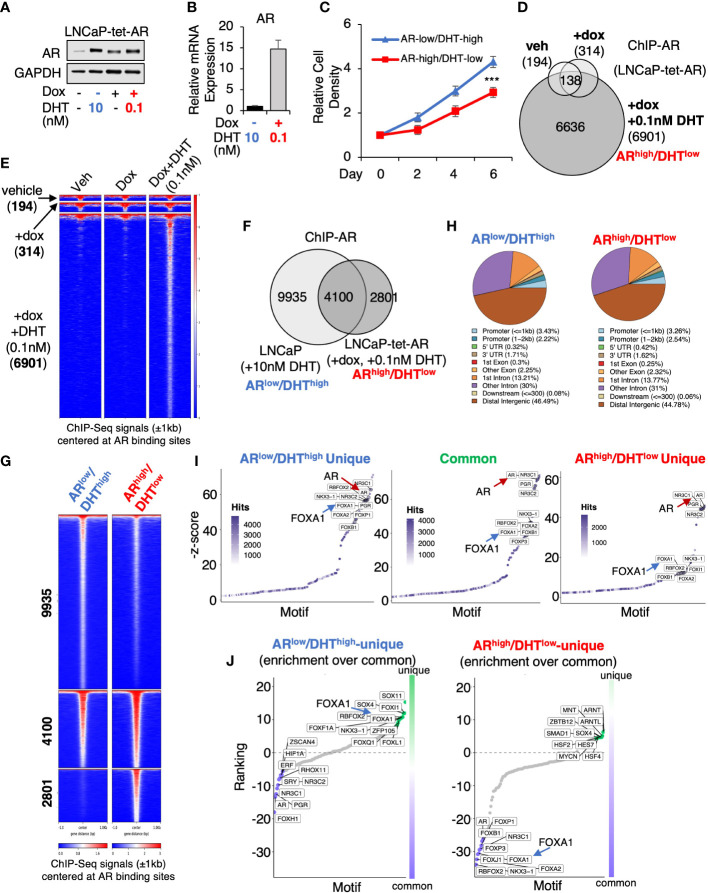
AR chromatin binding is altered in *AR* ^high^/DHT^low^ cells. **(A)** Immunoblotting for AR in LNCaP cells stably expressing tetracycline-regulated AR (LNCaP-tet-AR). **(B)** mRNA expression of AR in these cells (measured by qRT-PCR). **(C)** Proliferation of LNCaP-tet-AR cells treated with 10 nM DHT alone or with 0.25 µg/ml doxycycline plus 0.1 nM DHT for 0-6d. **(D)** Venn diagram for ChIP-AR peaks in LNCaP-tet-AR cells treated with vehicle, doxycycline alone, or doxycycline plus DHT (0.1nM for 4h). **(E)** Heatmap view for ChIP-seq signal intensity at these sites. **(F)** Venn diagram for ChIP-AR peaks in parental LNCaP cells treated with 10 nM DHT (*AR* ^low^/DHT^high^) and in LNCaP-tet-AR cells treated with 0.25 µg/ml doxycycline and 0.1 nM DHT (*AR* ^high^/DHT^low^). **(G)** Heatmap view for ChIP-seq signal intensity at clustered sites. **(H)** Genomic distribution for AR binding peaks. **(I)** Motif enrichment analysis for clustered AR binding sites (motifs were ranked based on z-score). **(J)** Unique motif enrichment by comparing motif enrichment for unique sites over common sites.

To determine whether AR overexpression in PCa cells stimulated by castrate levels of androgens can drive AR cistrome reprogramming, we generated an LNCaP PCa stable cell line (LNCaP-tet-AR) overexpressing tetracycline-regulated AR. As shown in **Figure 1A**, 0.1nM DHT (low-dose androgen) stimulation in cells with doxycycline treatment can stabilize AR protein to a similar level as 10nM DHT (high-dose androgen) stimulated AR in uninduced cells. However, the mRNA expression of AR is ~15-fold higher in the doxycycline-treated cells (**Figure 1B**). Therefore, we named these two conditions *AR* ^low^/DHT^high^ and *AR* ^high^/DHT^low^, which mimic the AR status under pre- and post-castration conditions, respectively. Cells can proliferate under both conditions but *AR* ^high^/DHT^low^ cells grew slightly slower (**Figure 1C**). We then performed ChIP-seq analyses of AR in LNCaP-tet-AR cells. As shown in **Figures 1D, E**, AR overexpression alone (without DHT stimulation) barely induced any AR binding (314 peaks), indicating that unliganded AR cannot bind to chromatin, whereas further stimulation with 0.1nM DHT can strongly induce AR chromatin binding (6,901 peaks). We then compared these AR binding sites (*AR* ^high^/DHT^low^ condition) with our previously published AR ChIP-seq in parental LNCaP cells stimulated with 10nM DHT (*AR* ^low^/DHT^high^ condition) (14,035 peaks, GSE114266). As shown in **Figures 1F, G**, while 4,100 AR binding peaks were conserved under both conditions, 2,801 new sites were distinctly detected in *AR* ^high^/DHT^low^ cells, indicating a reprogramming of AR cistrome despite that the genomic distribution of AR binding sites was similar under two conditions (**Figure 1H**). We next examined motif enrichment of transcription factors at these sites. As shown in **Figures 1I, J**, while AR binding motifs were commonly found at *AR* ^high^/DHT^low^-unique AR sites, the binding motifs of FOXA1, a critical pioneer factor of AR, was less enriched at these sites in comparison with *AR* ^low^/DHT^high^-unique or common sites, suggesting that *AR* ^high^/DHT^low^-unique AR binding may be less dependent on FOXA1. This observation is consistent with previous findings that loss of FOXA1 can trigger AR cistrome reprogramming in CRPC (24–26).

### AR activates a unique transcription program in *AR* ^high^/DHT^low^ cells

**Figure 2:**
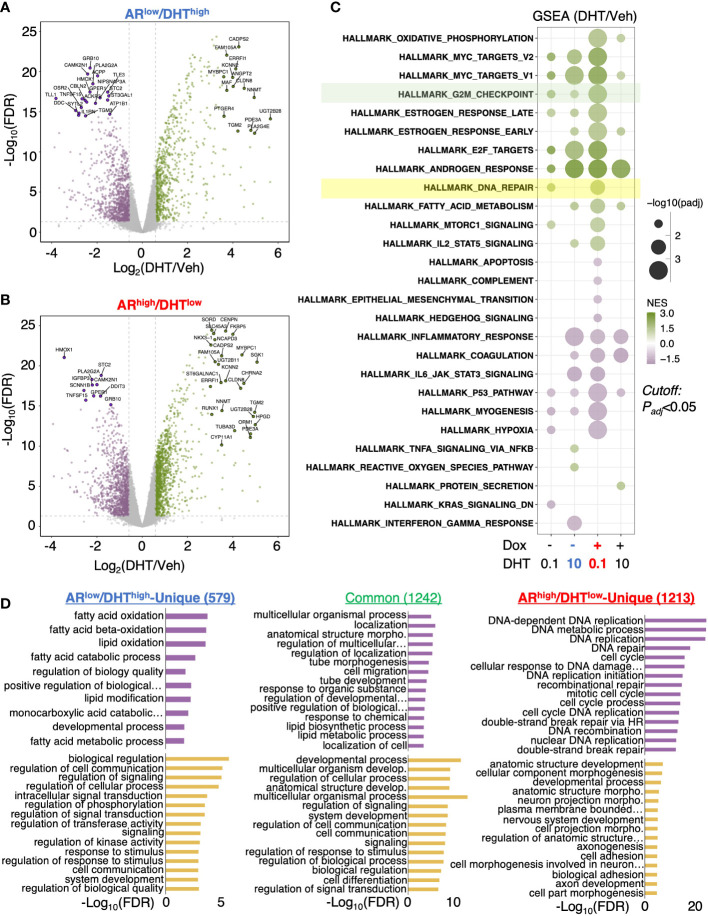
AR activates a unique transcription program in *AR* ^high^/DHT^low^ cells. **(A, B)** RNA-seq analyses were performed in LNCaP-tet-AR cells pretreated with or without doxycycline and then stimulated with 0, 0.1, or 10 nM DHT (for 24h). Volcano blots for DHT-regulated genes in cells treated with 10 nM DHT alone **(A)** or with 0.1 nM DHT plus doxycycline **(B)** were shown. **(C)** Gene Set Enrichment Analysis (GSEA) for enriched hallmark gene sets. **(D)** Gene Ontology analysis with the biological process (GO-BP) for differentially expressed genes (fold-change > 1.5; adjusted *P*-value < 0.05).

We next sought to determine the change of AR transcriptome in *AR* ^high^/DHT^low^ cells. A comprehensive RNA-seq analysis was performed in LNCaP-tet-AR cells under both *AR* ^low^/DHT^high^ and *AR* ^high^/DHT^low^ conditions, and DHT-upregulated and downregulated genes were identified (**Figures 2A, B**). We also included two other conditions for comparison, 0.1nM DHT only for modeling *AR* ^low^/DHT^low^ and 10nM DHT plus doxycycline for modeling *AR* ^high^/DHT^high^, although the latter condition is generally non-physiological. Gene Set Enrichment Analysis (GSEA) with hallmark gene set was conducted to provide a comprehensive view of AR transcriptomes under different conditions. As predicted, the androgen response pathway was enriched under all four conditions (**Figure 2C**). Interestingly, the G2M checkpoint pathway was noticeably more enriched in *AR* ^high^/DHT^low^ than *AR* ^low^/DHT^high^ cells, consistent with a previous finding that AR reprogramming in CRPC can distinctly activate the transcription of mitosis genes (27). More importantly, we also found that the DNA damage repair pathway was uniquely enriched in *AR* ^high^/DHT^low^ cells. This finding is consistent with a previous report showing that AR can regulate DNA repair genes in CRPC cells (28). Other *AR* ^high^/DHT^low^ uniquely enriched pathways include oxidative phosphorylation (metabolism) and MTORC1 signaling. In addition, AR also uniquely repressed several pathways including apoptosis, hypoxia, p53, and myogenesis in *AR* ^high^/DHT^low^ cells. By directly comparing androgen-regulated genes in *AR* ^low^/DHT^high^ versus *AR* ^high^/DHT^low^ cells, we identified 579 *AR* ^low^/DHT^high^-unique genes, 1,242 common genes, and 1,213 *AR* ^high^/DHT^low^-unique genes. Remarkably, the *AR* ^high^/DHT^low^-unique genes were highly enriched for DNA replication and damage repair functions (**Figure 2D**). Together, these data indicate that AR in CRPC cells can activate a distinct transcription program that is enriched for DNA damage repair functions.

### AR directly activates genes mediating DNA damage response

**Figure 3:**
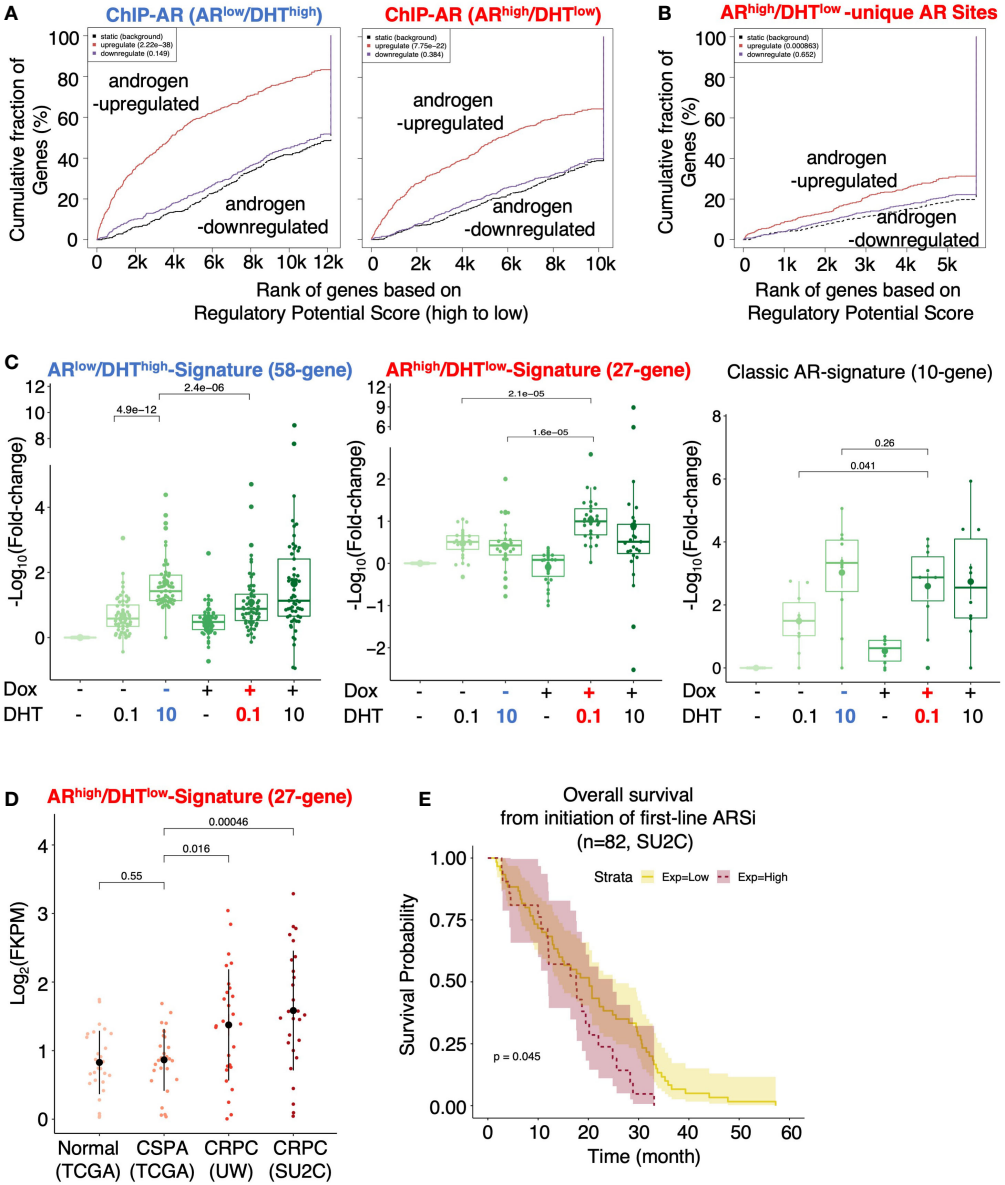
Identification of AR direct targets in *AR* ^high^/DHT^low^ cells. **(A, B)** Binding and Expression Target analysis (BETA) for the association of AR binding sites (ChIP-AR) and DHT-regulated genes in *AR* ^low^/DHT^high^ or *AR* ^high^/DHT^low^ cells **(A)** and the *AR* ^high^/DHT^low^-unique AR binding sites and DHT-regulated genes in *AR* ^high^/DHT^low^ cells **(B)**. **(C)** Boxplot for *AR* ^low^/DHT^high^ unique AR signature (58-gene), *AR* ^high^/DHT^low^ unique AR signature (27-gene), and previously published classic AR targets (10-gene) in LNCaP-tet-AR cells under indicated conditions. **(D)** The expression levels of *AR* ^high^/DHT^low^-unique AR signature in TCGA (normal and primary PCa), UW (mCRPC), and SU2C (mCRPC) datasets. **(E)** Kaplan-Meier survival analysis for the overall survival from the initiation of the first-line ARSi in mCRPC patients (SU2C cohort) with higher scores (purple, top 25%) of the signature versus the lower scores (yellow, bottom 75%).

We next combined the cistromic and transcriptomic analyses to identify the direct target genes of AR under both conditions. Using Binding and Expression Target Analysis (BETA) (17), we found that AR binding peaks were highly associated with androgen-upregulated genes but not androgen-repressed genes (**Figure 3A**), suggesting that the major activity of AR in these cells is activating transcription. The *AR* ^high^/DHT^low^ unique AR binding sites (2801) were also associated with AR-activated genes, suggesting that these AR bindings are transcriptionally active (**Figure 3B**). We then developed two AR target signatures to represent unique AR activities under *AR* ^low^/DHT^high^ and *AR* ^high^/DHT^low^ conditions. While the *AR* ^low^/DHT^high^ signature (58-gene) had a similar expression pattern as the previously reported classic AR target signature (10-gene) (29), the *AR* ^high^/DHT^low^ signature (27-gene) was clearly regulated in a different pattern (**Figure 3C**). We next examined whether this *AR* ^high^/DHT^low^ signature is upregulated in CRPC clinical cohorts. As shown in **Figure 3D**, the expression of this signature was significantly higher in two publicly available metastatic CRPC cohorts (UW, SU2C) in comparison with the normal or primary PCa cohort (TCGA) (7, 29–33). More importantly, this signature was associated with poorer clinical outcomes in response to the first-line ARSi treatments (**Figure 3E**).

**Figure 4:**
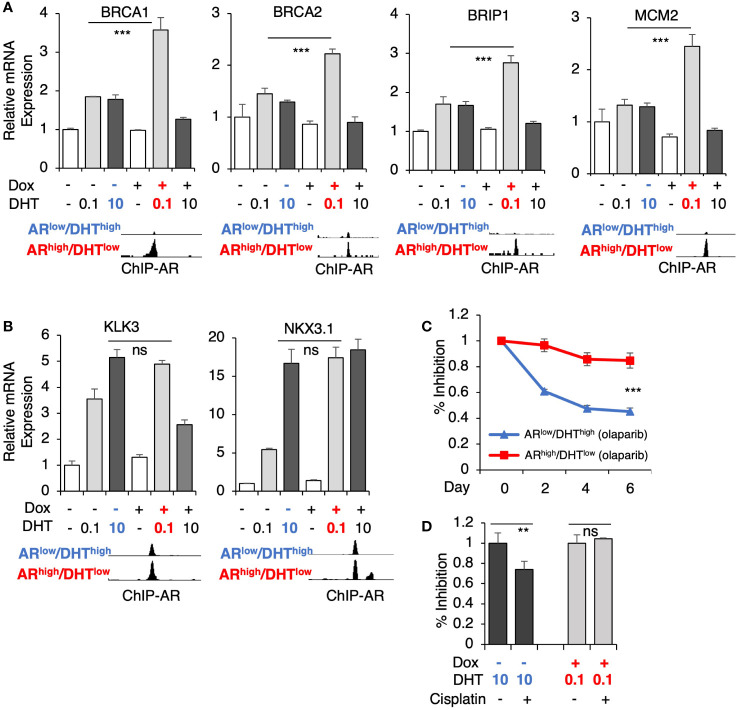
AR directly activates genes mediating DNA damage response. **(A, B)** LNCaP-tet-AR cells were pre-treated with or without 0.25 µg/ml doxycycline for 2d and then stimulated by DHT (0.1 or 10 nM for 24h). mRNA expression of a panel of *AR* ^high^/DHT^low^ uniquely regulated genes **(A)** and classic AR-targeted genes **(B)** were measured. A genome view of AR binding peaks at the target gene loci was also shown for each gene. **(C, D)** Cell growth inhibition for LNCaP-tet-AR cells under the indicated conditions and treated with or without 10 µM olaparib for 0-6d **(C)**, or 50 µM cisplatin for 2d **(D)**.

Several genes for DNA replication and damage repair pathways were selected for the subsequent validation, including *BRCA1*, *BRCA2*, *BRIP1* (BRCA1 interacting helicase 1), and *MCM2* (subunit of DNA helicase protein), which all harbor unique AR binding sites in *AR* ^high^/DHT^low^ cells (**Figure 4A**). Consistent with the RNA-seq results, DHT treatment alone only modestly increased the expression of these targets whereas the combination of low-dose DHT with AR overexpression can significantly induce their expression. These results are in sharp contrast to the classic AR-regulated genes (*KLK3* and *NKX3.1*) which are similarly induced under both *AR* ^low^/DHT^high^ and *AR* ^high^/DHT^low^ conditions (**Figure 4B**). Next, we examined the responses of these cells to an FDA-approved PARP inhibitor, olaparib (34, 35), which is a DNA damaging agent. As shown in **Figure 4C**, the olaparib treatment effectively suppressed the growth of *AR* ^low^/DHT^high^ cells, but had a much weaker effect on the growth of *AR* ^high^/DHT^low^ cells, indicating that *AR* overexpression with decreased androgens can impair PCa cell response to genotoxic stress. A similar result was also obtained for the response to cisplatin treatments (**Figure 4D**).

### EZH2 is engaged in the reprogramming of AR

**Figure 5:**
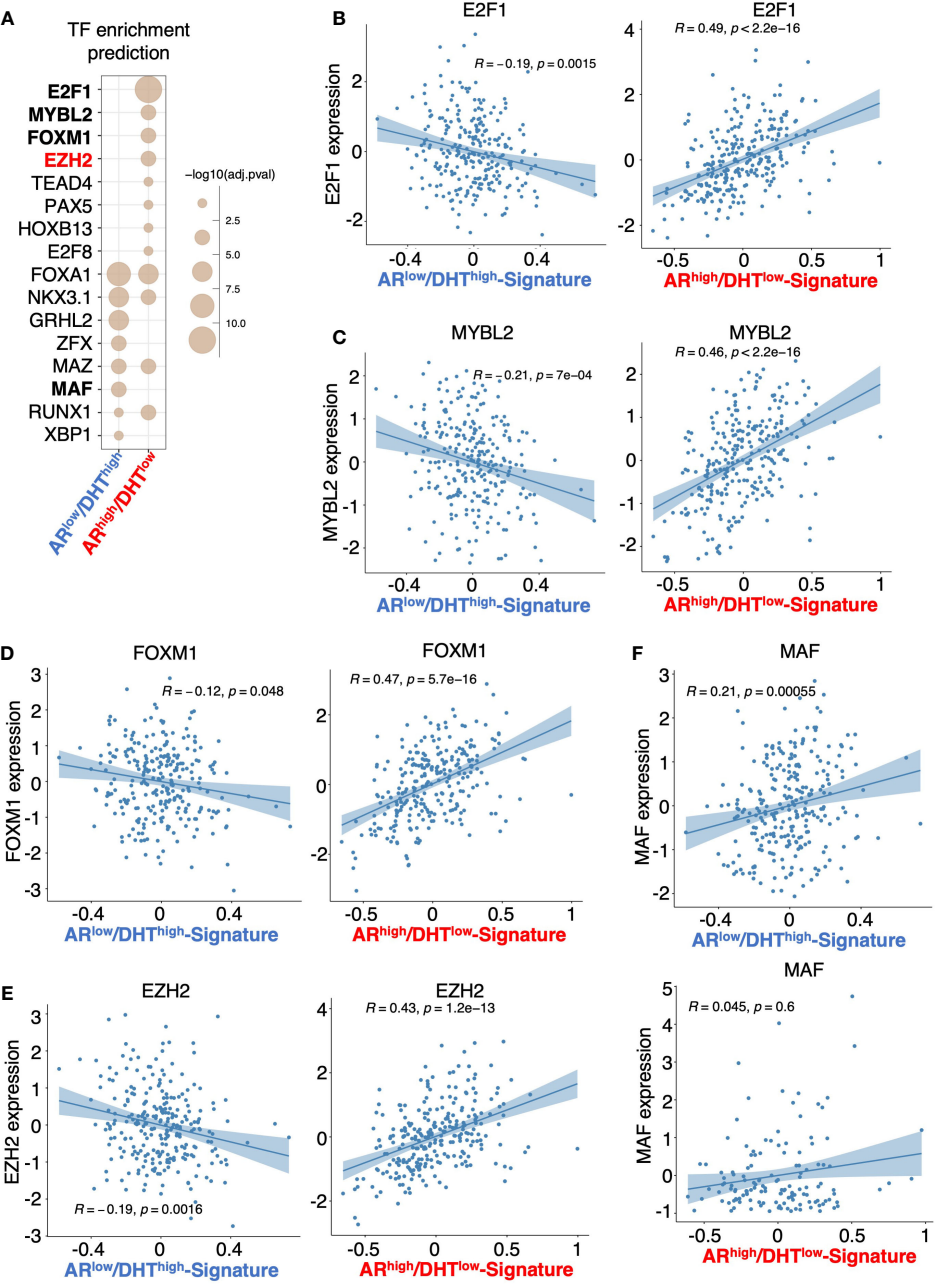
Prediction of transcription or epigenetic factors involved in the reprogramming of AR. **(A)** Prediction of the involvement of transcription factor/chromatin regulator in mediating AR activity in *AR* ^low^/DHT^high^ cells or *AR* ^high^/DHT^low^ cells using Causal Inference Enrichment (CIE) platform for significant gene expression (fold-change > 2, FDR < 0.05). ChIP-Atlas prostate cell line dataset was selected as the database, and Fisher’s exact test was used as the enrichment method. Enriched factors with adjusted *P*-value < 0.05 are shown. **(B–F)** Correlation of predicted factors, E2F1 **(B)**, MYBL2 **(C)**, FOXM1 **(D)**, EZH2 **(E)**, and MAF **(F)**, with the expression of *AR* ^low^/DHT^high^ signature or *AR* ^high^/DHT^low^ signature in mCRPC patient dataset.

Epigenetic factors, particularly histone modifiers such as LSD1 and EZH2 (11, 12, 36, 37), can strongly influence AR activity and thus participate in the reprogramming of AR signaling in CRPC. Therefore, we next sought to identify epigenetic factors that may contribute to the distinct activity of AR in *AR* ^high^/DHT^low^ cells. We have recently developed an integrated platform, CIE, for the identification and interpretation of active regulators of transcriptional response based on publicly available ChIP-seq data with tissue-specific gene expression data (23). Using this tool, we can then predict the regulation of potential transcription factors and epigenetic regulators based on identified AR transcriptomes. As shown in **Figure 5A**, several transcription or epigenetic factors were predicted to distinctly regulate the AR transcription program in *AR* ^high^/DHT^low^ cells, including E2F1, MYBL2, FOXM1, and EZH2. Consistent with the prediction, the expression levels of these factors were strongly and positively correlated with *AR* ^high^/DHT^low^ signature in mCRPC dataset (SU2C) but negatively associated with *AR* ^low^/DHT^high^ signature (**Figures 5B–E**). On the contrary, the expression of MAF, which was predicted as *AR* ^low^/DHT^high^ specific factors, was not associated with *AR* ^high^/DHT^low^ signature in mCRPC samples (**Figure 5F**).

**Figure 6:**
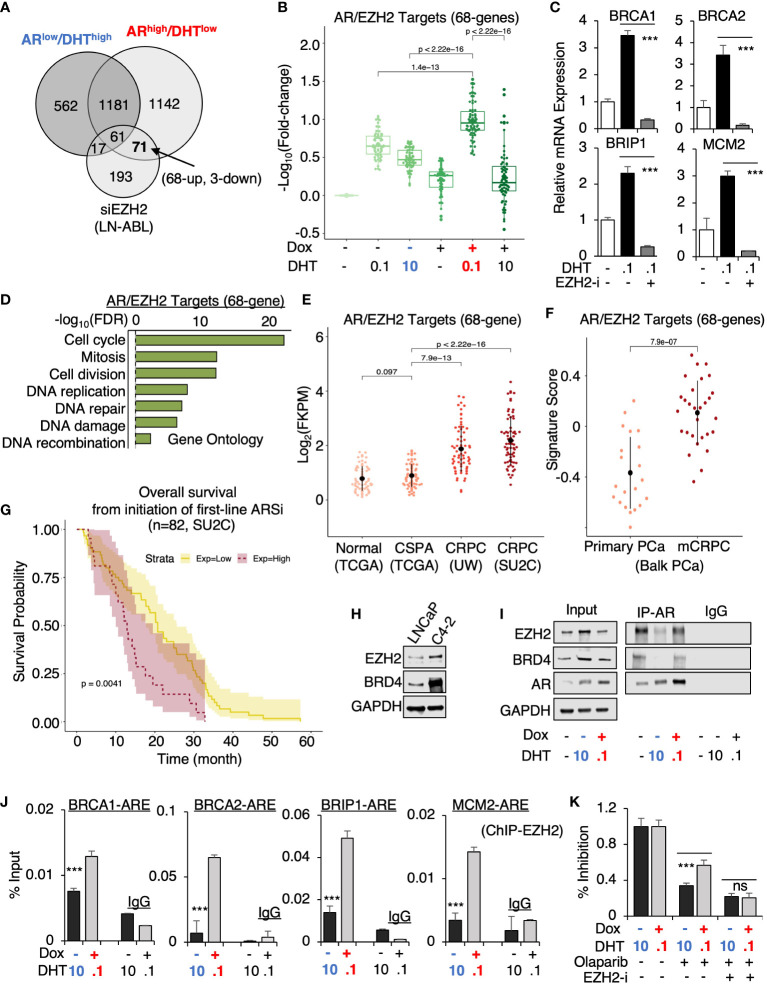
EZH2 is engaged in the reprogramming of AR in CRPC. **(A)** Venn diagram for DHT-regulated genes in *AR* ^low^/DHT^high^ and *AR* ^high^/DHT^low^ LNCaP-tet-AR cells and EZH2-regulated genes in LNCaP-ABL cells (fold-change > 1.5, adjusted *P*-value < 0.05). **(B)** Boxplot for AR/EZH2 co-target gene expression (68-genes) in LNCaP-tet-AR cells under indicated conditions. **(C)** qRT-PCR for *AR* ^high^/DHT^low^-unique AR regulated genes treated with 0.1 nM DHT and an EZH2 inhibitor, GSK126 (50μM for 24h). **(D)** Gene ontology of AR/EZH2 co-regulated 68 genes (FDR< 0.05). **(E, F)** AR/EZH2 co-target gene expression (68-gene) in TCGA, UW, and SU2C datasets **(E)** and Balk PCa dataset **(F)**. **(G)** Kaplan-Meier survival analysis for the overall survival from the initiation of the first-line ARSi in mCRPC patients (SU2C cohort) with higher scores (purple, top 25%) of AR/EZH2 target signatures versus the lower scores (yellow, bottom 75%). **(H)** Immunoblotting for indicated proteins in LNCaP versus LNCaP-C4-2 cells. **(I)** Immunoblotting for indicated proteins in LNCaP-tet-AR cells immunoprecipitated with AR. **(J)** ChIP-qPCR of EZH2 at indicated AR binding sites. **(K)** Cell growth inhibition for LNCaP-tet-AR cells under the indicated conditions and treated with 30µM olaparib for 2d, or olaparib plus GSK126 (50 µM for 2d).

From this prediction analysis, we identified EZH2 as the major epigenetic factor that is potentially involved in AR reprogramming in CRPC. Using a published EZH2 target profiling dataset (in an LNCaP-derived CRPC line, LNCaP-ABL, GSE39452), we found that 71 genes were possibly co-targeted by EZH2 and AR in *AR* ^high^/DHT^low^ cells but only 17 genes were co-targeted in *AR* ^low^/DHT^high^ cells (**Figure 6A**). Within this 71 gene subset, 68 of them were androgen-upregulated and 3 of them were downregulated and the expression of these 68 genes was more significantly activated in *AR* ^high^/DHT^low^ cells (**Figure 6B**). The four identified *AR* ^high^/DHT^low^-unique AR-regulated genes were also found in this subset and the DHT-induced expression of these genes can be suppressed by EZH2 inhibitor treatment (**Figure 6C**). Examining the function enrichment of this 68-gene subset using the gene ontology analysis, we found that these genes were highly enriched for cell cycle regulation and DNA replication and damage repair (**Figure 6D**). Next, we examined the clinical relevance of this AR/EZH2 co-targets signature. As shown in **Figure 6E**, the expression of AR/EZH2 co-targets was significantly higher in two mCRPC cohorts than in the primary PCa cohort. A similar result was also obtained using Balk PCa dataset (**Figure 6F**), which contains both primary PCa and CRPC samples (34, 35). Moreover, we also found that this set of AR/EZH2 co-targets were strongly associated (*P*=0.0041) with poor clinical outcomes in response to ARSi treatments (**Figure 6G**).

Finally, we examined the potential mechanism for this unique involvement of EZH2 in mediating AR activity. The expression of EZH2 and another epigenetic factor BRD4, which is associated with enhancer activation and can function as an AR coactivator through interaction with the acetylated histones (38), were both elevated in LNCaP-derived CRPC cells (LNCaP-C4-2) (**Figure 6H**). Interestingly, we can detect strong interaction of AR and EZH2 at the basal condition (with minimal androgens) (**Figure 6I**). However, this interaction was markedly impaired under *AR* ^low^/DHT^high^ condition but recovered under *AR* ^high^/DHT^low^ condition, suggesting that EZH2 may enhance AR transcription program preferentially in CRPC but not in primary PCa. Interestingly, AR-BRD4 interaction also showed a similar pattern as EZH2. Consistent with the protein-protein interaction results, EZH2 chromatin binding at those gained AR binding sites was markedly higher in *AR* ^high^/DHT^low^ cells than in *AR* ^low^/DHT^high^ cells (**Figure 6J**). Moreover, we examined whether EZH2 is involved in mediating CRPC tumor cell response to DNA damage. As shown in **Figure 6K**, EZH2 inhibition resensitized *AR* ^high^/DHT^low^ cells to the treatment of olaparib, suggesting that EZH2 inhibitor and DNA damaging treatments can be combined to treat CRPC. Together, these data provide strong evidence for the collaboration of EZH2 in the reprogramming of AR signaling, particularly on the transcriptional activation of DNA replication and damage repair genes in CRPC, and suggest that the presence of low-dose androgen in CRPC may be a key factor for maintaining the interaction between AR and EZH2.

## Discussion

It is well documented that AR signaling is intensively reprogrammed in CRPC (27). One possible mechanism is the emergence of ligand-binding domain truncated and constitutively activated AR splice variants, such as AR-V7, which cannot be targeted by common AR antagonists (39–42). Other mechanisms, such as changes in AR pioneer factors, cofactors, and epigenetic factors, also likely contribute to the reprogramming (12, 24–26, 36, 38, 43). While the overexpression of full-length AR in CRPC is known to resensitize AR signaling to the low levels of androgens, it is not entirely clear whether *AR* gene overexpression alone in CRPC can lead to significant transcriptional reprogramming. In addition to the change in *AR* levels, another key component regulating the AR activity in CRPC is the presence of a much lower level of androgens in castrated tumors. Therefore, in this study, we designed a model to rapidly switch between the pre-castration (*AR* ^low^/DHT^high^) and post-castration (*AR* ^high^/DHT^low^) AR status and then examined whether the combination of *AR* overexpression with low levels of ligands may significantly alter AR transcription program. Our study clearly indicates that PCa cells switching from *AR* ^low^/DHT^high^ to *AR* ^high^/DHT^low^ status can acutely and dramatically alter AR chromatin binding and transcription outputs. More importantly, we fully characterized this AR reprogramming and revealed that one critical outcome is the distinct activation of genes mediating DNA replication and damage repair pathways, which may allow CRPC cells to become resistant to genotoxic stress. Interestingly, our previous studies indicated that the combination of AR overexpression and high-dose androgens can transcriptionally suppress DNA replication genes, which provides a molecular basis for the high-dose testosterone treatment in CRPC (5, 44, 45). Although we did not focus on this non-physiological condition in this study, our data showed that the activation of DNA damage repair genes almost disappeared under *AR* ^high^/DHT^high^ condition (see **Figures 2C** and **6B**), which may be contributed by this transcriptional repression mechanism. Overall, our study provides new molecular insights into the mechanism of AR signaling reprogramming during PCa progression and suggests that AR overexpression alone in combination with the castrate level of androgens is a driving force of rapid AR signaling reprogramming in CRPC.

Epigenetic changes are key factors for the PCa cells adapted to the AR signaling inhibition treatment. Using our recently developed bioinformatic tool, we predicted the positive involvement of EZH2 in regulating the AR transcriptome under *AR* ^high^/DHT^low^ conditions and identified AR/EZH2 co-targeting genes. Previous studies have demonstrated EZH2 as a critical AR coregulator through its activator function, which is independent of its H3K27 methyltransferase activity (36, 46). However, whether EZH2 is engaged in the reprogramming of AR signaling in CRPC is not clear. Interestingly, these AR/EZH2 co-targeting genes are highly enriched for DNA replication and damage repair pathways, and higher expression of these genes in CRPC is associated with worse outcomes in response to ARSi. This finding is consistent with a recent report which showed EZH2 can drive tumor resistance to DNA-damaging treatments in CRPC (13). Moreover, from our co-IP study, we also revealed an interesting finding that AR-EZH2 interaction is only maintained under minimal or low-dose androgen conditions but is markedly impaired by high levels of androgens. Although the molecular basis behind this observation remains to be determined in the future, it explains why EZH2 can preferentially enhance AR transcription program in CRPC. Therapeutically, the FDA-approved EZH2 inhibitor, tazemetostat (47, 48), is being tested in the combination with AR signaling inhibition agents to treat metastatic CRPC (e.g., CELLO-1 trial). More importantly, the findings from us and other groups clearly support the rationale for testing EZH2 inhibitors in combination with DNA-damaging agents, such as FDA-approved PARP inhibitors, in CRPC patients (e.g., NCT04846478).

## Data availability statement

The datasets presented in this study can be found in online repositories. The names of the repository/repositories and accession number(s) can be found below: Gene Expression Omnibus, series number: GSE211641.

## Author contributions

ML, DH, and CC designed the study. ML, MQL, LT, BK, SZ, SG, SP, JM, KZ, and DH performed experiments and analyzed the results. ML, DH, and CC wrote the manuscript. All authors contributed to the article and approved the submitted version.

## Funding

This work is supported by grants from NIH (R01 CA211350 to CC and U54 CA156734 to JM and CC), DOD (W81XWH-19-1-0361 and W81XWH-21-1-0267 to CC, W81XWH-19-1-0777 to SG). CC and DH were supported by Proposal Development Award from University of Massachusetts Boston. ML and SZ were supported by the graduate fellowship from the Integrative Biosciences/Computational Sciences Programs at the University of Massachusetts Boston. LT and BK were supported by University of Massachusetts Boston/Dana-Farber Cancer Institute Partnership Summer Program.

## Conflict of interest

The authors declare that the research was conducted in the absence of any commercial or financial relationships that could be construed as a potential conflict of interest.

## Publisher’s note

All claims expressed in this article are solely those of the authors and do not necessarily represent those of their affiliated organizations, or those of the publisher, the editors and the reviewers. Any product that may be evaluated in this article, or claim that may be made by its manufacturer, is not guaranteed or endorsed by the publisher.
